# Radiation-triggered acute exacerbation of progressive fibrotic interstitial lung diseases: ‘Are we advancing the frontier or crossing a dangerous line?’

**DOI:** 10.1177/17534666261446877

**Published:** 2026-05-05

**Authors:** Ilias E. Dimeas, Angeliki Miziou, Patrick D. Mitchell, Zoe Daniil, Cormac McCarthy

**Affiliations:** School of Medicine, University College Dublin, Dublin 4, Ireland; Department of Respiratory Medicine, St. Vincent’s University Hospital, Dublin, Ireland; Respiratory Department, Tallaght University Hospital, Dublin, Ireland; Department of Respiratory Medicine, Faculty of Medicine, University of Thessaly, Biopolis, Larissa, Greece; Department of Respiratory Medicine, Faculty of Medicine, University of Thessaly, Biopolis, Larissa, Greece; Respiratory Department, Tallaght University Hospital, Dublin, Ireland; School of Medicine, Trinity College Dublin, Dublin, Ireland; Department of Respiratory Medicine, Faculty of Medicine, University of Thessaly, Biopolis, Larissa, Greece; School of Medicine, University College Dublin, Dublin, Ireland; Department of Respiratory Medicine, St. Vincent’s University Hospital, Dublin, Ireland

**Keywords:** acute exacerbation, antifibrotic therapy, fibrotic interstitial lung disease, prophylactic steroids, radiation pneumonitis, stereotactic ablative radiotherapy

## Abstract

Fibrotic interstitial lung diseases (F-ILDs) are increasingly complicated by early-stage non-small cell lung cancer. For many of these patients, stereotactic ablative body radiotherapy (SABR) is the only feasible curative-intent option. However, pre-existing fibrosis markedly increases the risk of severe, sometimes fatal, post-treatment respiratory events. These episodes are usually labelled radiation pneumonitis (RP), yet their timing, distribution and lethality often resemble acute exacerbations (AEs) of the underlying interstitial lung disease (ILD) rather than classical, field-confined RP. In this narrative review, we synthesise observational data from the SABR series enriched for ILD, focusing on acute respiratory deterioration within weeks to months after treatment. We examine how inconsistent terminology and heterogeneous case definitions obscure the distinction between RP and radiation-triggered AE (RT-AE)-ILD, and we summarise clinical, radiological, physiological, dosimetric and biomarker risk factors. Mechanistic models suggest that radiation may amplify a primed fibrotic lung via convergent epithelial, endothelial and immune pathways, with potential modulation by antifibrotic therapy, corticosteroid exposure and infection. We argue that in patients with progressive F-ILD, at least a subset of post-SABR ‘pneumonitis’ episodes are better conceptualised as RT-AE, with implications for corticosteroid stewardship, infection prophylaxis and continuation of antifibrotics. Given the absence of trial-level evidence and the high baseline mortality of F-ILD, management must prioritise multidisciplinary evaluation, explicit shared decision-making and cautious individualisation rather than reflexive transplantation of RP algorithms. Prospective registries, standardised ILD phenotyping and translational studies are urgently needed to define risk boundaries and test whether antifibrotic-radiation strategies or particle therapies can safely deliver curative-intent treatment to this highly vulnerable population.

## Introduction and clinical rationale

Fibrotic interstitial lung diseases (F-ILDs) such as idiopathic pulmonary fibrosis (IPF), fibrotic hypersensitivity pneumonitis (F-HP) and connective-tissue-disease-associated interstitial lung diseases (CTD-ILDs) share a relentlessly progressive course and limited therapeutic options. Among these, lung cancer arises with disproportionate frequency, reflecting shared profibrotic pathways, smoking exposure and repeated epithelial injury.^1^ For many affected patients, surgical resection is contraindicated by severely reduced pulmonary reserve or diffusion capacity, leaving stereotactic ablative body radiotherapy (SABR) as the only curative-intent modality. SABR achieves local-control rates comparable to surgery in early-stage non-small cell lung cancer (NSCLC), yet its safety profile in fibrotic lungs remains uncertain.^2^ In patients with underlying interstitial lung disease (ILD), post-radiation respiratory deterioration, often radiologically diffuse and clinically fulminant, carries a mortality that can surpass that of the tumour itself.^3^ These events have historically been labelled ‘radiation pneumonitis’, but mounting evidence suggests that, in fibrotic hosts, they may represent radiation-triggered acute exacerbations (RT-AEs) of the underlying disease rather than classic radiation injury.^4^ Distinguishing these overlapping entities is clinically crucial yet diagnostically elusive. The implications extend beyond nomenclature. Standard radiation-oncology practice often includes prophylactic or empiric corticosteroids to mitigate pneumonitis risk; however, in IPF and other progressive F-ILDs (PF-ILDs), corticosteroids have repeatedly failed to show survival benefit and may predispose to infection and secondary immune paralysis.^5,6^ This tension between oncologic benefit and pulmonary fragility leaves clinicians without evidence-based guidance. Despite the growing recognition of triggered acute exacerbations (AEs) following surgery, infection or drug exposure, the phenomenon following SABR has received comparatively little formal attention. Available data derive largely from small, retrospective series, predominantly from East Asian cohorts, with heterogeneous case definitions and variable dosimetric reporting.^7,8^ Nevertheless, across studies, the signal is consistent: pre-existing fibrotic change, higher lung-dose volumes and central tumour location are associated with markedly increased risk. Accordingly, most clinical series and reviews of SABR in patients with ILD have framed severe post-treatment respiratory deterioration as radiation pneumonitis (RP), often extrapolating dose-volume concepts from non-fibrotic lungs. However, in patients with PF-ILDs, a subset of these events differs in timing, radiological distribution, severity and outcome from classical RP. Rather than isolated radiation toxicity, they may represent RT-AEs of the underlying fibrotic disease, driven predominantly by host susceptibility. Explicitly separating between these overlapping but biologically distinct processes carries direct implications for corticosteroid use, antifibrotic therapy continuation, infection risk and multidisciplinary treatment planning. The objective of this review is therefore to synthesise the available clinical, radiologic and mechanistic evidence regarding acute respiratory deterioration after SABR in F-ILDs, explore the hypothesis that these events constitute RT-AEs rather than conventional RP, and highlight the implications for antifibrotic therapy, corticosteroid stewardship and future translational research. By integrating perspectives from pulmonology, radiation oncology and immunopathology, this narrative review aims to propose the conceptual framework of RT-AE as a distinct clinical phenotype in F-ILD following SABR and to prompt systematic investigation into an under-recognised but potentially preventable cause of respiratory death in progressive fibrotic lung disease.

## Methods

This narrative review was conducted in accordance with the Scale for the Assessment of Narrative Review Articles (SANRA) guideline (Supplemental Material).^9^ A non-systematic literature search was performed in PubMed, Embase and Web of Science to identify relevant English-language publications from 2000 to 2025. Search terms included combinations of ‘fibrotic interstitial lung disease’, ‘stereotactic ablative radiotherapy’, ‘acute exacerbation’, ‘radiation pneumonitis’, ‘antifibrotic therapy’ and ‘prophylactic steroids’. An example PubMed query was: (interstitial lung disease OR idiopathic pulmonary fibrosis OR fibrotic) AND (stereotactic body radiotherapy OR SBRT OR SABR) AND (acute exacerbation OR radiation pneumonitis); terms were adapted for Embase and Web of Science. Approximately 200 records were screened at the title/abstract level across databases (after de-duplication), 80 full-text articles were assessed and 68 publications were prioritised for inclusion in the narrative synthesis and cited in the reference list. Studies were selected based on clinical relevance and their contribution to understanding radiation-triggered respiratory deterioration in F-ILDs, with emphasis on ILD-specific SABR outcomes, AE-ILD definitions, dosimetric predictors, antifibrotic interactions and infection risk. No formal inclusion or exclusion criteria, Preferred Reporting Items for Systematic Reviews and Meta-Analyses (PRISMA) methodology or quantitative risk-of-bias assessment were applied, consistent with the narrative and hypothesis-generating nature of the review.

### Definitions and diagnostic ambiguity

The terminology describing acute respiratory deterioration after SABR in patients with F-ILD remains inconsistent and conceptually problematic. Although the same technique is often termed stereotactic body radiation therapy (SBRT) in the oncologic literature, the designation SABR is used throughout this review, as it better conveys the biologically ablative intent rather than the technical delivery method.^10,11^ In clinical reporting, such events are most often documented as RP, yet their radiologic distribution, temporal evolution and clinical lethality frequently overlap with AE of the underlying fibrotic process.^3,8,12,13^ This distinction is more than semantic, as it directly influences corticosteroid use, antifibrotic continuation, infection surveillance and decisions regarding retreatment.^5,6^ The 2016 International Working Group Report on Acute exacerbation of idiopathic pulmonary fibrosis (AE-IPF) provided a modern framework by redefining AE as an acute, clinically significant respiratory deterioration characterised by new, bilateral ground-glass opacities or consolidation superimposed on established fibrosis, in the absence of alternative explanations such as heart failure or fluid overload. Crucially, it introduced the category of triggered AEs, precipitated by infection, drug toxicity or surgical or procedural stress, alongside idiopathic forms. Within this taxonomy, radiation exposure constitutes a plausible trigger. Several subsequent analyses have applied this concept beyond IPF to other PF-ILDs, including F-HP and CTD-ILDs.^13,14^ By contrast, classical RP represents a dose-dependent inflammatory reaction confined to the irradiated field, typically emerging 1–6 months after treatment and radiographically demarcated by the isodose contour. Histologically, it is dominated by alveolar epithelial injury, lymphocytic infiltration and type II pneumocyte hyperplasia. In patients without ILD, RP usually remains self-limited or responds to short corticosteroid courses.^15,16^ In fibrotic lungs, however, this paradigm breaks down. Multiple retrospective series have documented fulminant, bilateral, and often fatal pneumonitis after SABR in ILD patients, frequently developing within mere weeks of therapy and extending beyond the high-dose field.^7,8,17^ Pathologic observations reinforce this overlap. Autopsy studies in AE-IPF reveal diffuse alveolar damage with hyaline membranes and organising fibroblast foci, a pattern indistinguishable from the diffuse form of RP at high-dose levels.^18^ Similarly, reports of organising pneumonia arising after SABR describe lesions extending beyond the irradiated volume and occurring in otherwise stable fibrotic lungs.^19^ The timing, distribution and histology therefore suggest that radiation may serve not only as a local tissue injury but also as a systemic trigger amplifying the fibro-inflammatory milieu characteristic of PF-ILD. Clinically, these events defy neat categorisation. In some patients the opacity pattern remains peri-field and subacute, consistent with classical RP; in others, diffuse ground-glass changes appear within 2–4 weeks, rapidly progressing to respiratory failure and death despite corticosteroids, aligning more closely with the AE-ILD phenotype described after surgery or infection.^20–22^ Dosimetric studies show that these outcomes often occur even when lung V20, the percentage of total lung volume receiving at least 20 Gy of radiation, and mean lung dose (MLD) remain below accepted safety thresholds,^23,24^ reinforcing the notion that host vulnerability rather than dose distribution alone determines risk. These dose-volume parameters follow the Vx convention, indicating the proportion of lung receiving at least x Gy (e.g. V5, V20, V25), and are widely used in the radiation oncology literature. Because lung biopsy is rarely feasible in this setting, most cases are classified retrospectively by expert consensus using a combination of radiographic pattern, temporal association and therapeutic response. Even so, inter-observer agreement remains poor, and published series often use ‘radiation pneumonitis’, ‘acute exacerbation’ and ‘acute interstitial pneumonia’ interchangeably.^25,26^ The resultant heterogeneity complicates meta-analysis and obscures mechanistic inference. For the purposes of this review, the expression ‘radiation-triggered acute exacerbation (RT-AE)’ is used descriptively to denote any acute, non-infectious respiratory deterioration occurring within weeks of SABR in a patient with PF-ILD, in whom the extent or rapidity of parenchymal involvement exceeds what would be expected for conventional RP. This terminology does not constitute a new diagnostic entity but rather a conceptual framework acknowledging the biological continuum between focal radiation injury and systemically amplified AE-ILD. Recognising this ambiguity is essential: therapeutic reflexes derived from classical RP, particularly early high-dose corticosteroids, may prove harmful in this vulnerable population, whereas precision in diagnosis could guide antifibrotic continuation, infection prophylaxis and research toward mechanistic clarity. To clarify the key clinical and biological distinctions between classical RP and RT-AE in patients with F-ILD, the principal differentiating features are summarised in Table 1.

**Table 1.** Comparative features of classical RP and RT-AE in patients with F-ILDs.

Feature	Classical RP	RT-AE
Timing of onset	Typically occurs weeks to a few months after radiotherapy	May occur early or in the subacute period after SABR, sometimes with abrupt clinical deterioration
Radiological distribution	Predominantly confined to the irradiated lung region	Often diffuse or bilateral, extending beyond the radiation field
Relationship to radiation field	Closely related to the treated volume and dose distribution	Often poorly confined to the radiation field; injury may involve non-irradiated lung
Dose dependency	Classically associated with lung dose-volume parameters	Less clearly dose-dependent; risk appears modulated by underlying fibrotic lung disease
Response to corticosteroids	Frequently responsive to corticosteroid therapy	Response often limited or unpredictable; corticosteroids may confer limited benefit
Mortality	Variable, generally lower than tumour-related mortality in early-stage disease	Often severe and associated with high short-term mortality
Dominant pathophysiological driver	Local radiation-induced lung injury	Host-driven vulnerability of fibrotic lung, with radiation acting as a trigger

F-ILD, fibrotic interstitial lung disease; RP, radiation pneumonitis; RT-AE, radiation-triggered acute exacerbation; SABR, stereotactic ablative body radiotherapy.

### Risk of acute respiratory events after SABR in PF-ILDs

Acute respiratory toxicity after SABR varies widely across patient populations, and the risk is disproportionately elevated in those with pre-existing interstitial lung abnormalities. Barriger et al.,^17^ in a retrospective study of 251 patients treated with SABR, reported an overall rate of Grade 2–4 RP of 9.4%. In a Japanese survey,^27^ treatment-related deaths after SABR for pulmonary lesions occurred in 0.46% of patients, whereas in the study by Onishi et al.,^8^ fatal RP was observed in 1.3%, likely reflecting differences in baseline patient characteristics. Notably, in the latter cohort, 63% of patients who developed fatal RP showed interstitial pneumonia on computed tomography (CT).

Across multiple studies, patients with ILD undergoing SABR exhibit substantially higher rates of RP and worse overall survival (OS) compared with non-ILD patients. Grade ⩾3 RP occurs in approximately 10%–40% of patients with pulmonary interstitial changes, with fatal events (Grade 5) reported in more than 5%, and rates may be higher in prospective studies.^8^ Vuong et al.^28^ reported a cumulative Grade ⩾2 RP rate of 16.1% in ILD patients versus 3.6% in non-ILD patients, accompanied by reduced median OS (36.1 vs 54.3 months). In a retrospective analysis by Okubo et al.^29^ of 71 primary or metastatic lung tumours treated with SABR, Grade 2–5 RP was observed in 5 of the 11 ILD cases (45%): Grade 2 in 2 patients, Grade 3 in 1 and Grade 5 in 2. Ueki et al.^30^ similarly found pre-existing ILD to be a significant risk factor for symptomatic and severe RP at 1 year (⩾Grade 2 RP: 55.0% vs 13.3%; ⩾Grade 3 RP: 10.0% vs 1.5%). Yoshitake et al.^22^ reported higher rates of ⩾Grade 2 RP and poorer 2-year OS in patients with interstitial changes (44.4% vs 4.1% RP; 49.6% vs 86.7% OS). Tsurugai et al.^31^ demonstrated increased Grade 3 RP in patients with idiopathic interstitial pneumonias, particularly those with usual interstitial pneumonia (UIP) or combined pulmonary fibrosis and emphysema, along with significantly lower 2-year OS (42.2% vs 80.9%). Bahig et al.,^12^ in a retrospective analysis of 504 stage I lung cancer patients treated with SABR, identified a 4% rate of ⩾Grade 3 RP overall but a markedly higher incidence in ILD patients (32% in ILD-positive vs 2% in ILD-negative, *p* < 0.001), with 21% of ILD-positive patients developing Grade 5 RP. Chen et al.^3^ showed that patients with IPF experienced substantially higher SABR-related mortality and toxicity (33% and 71% vs 14% and 22%, respectively) as well as lower 3-year OS (48.8% vs 68.9%). Glick et al.^7^ reported roughly threefold higher RP rates in ILD patients (Grade 2: 20.5% vs 5.8%; Grade 3: 10.3% vs 1.0%), although without a corresponding OS difference. Yamaguchi et al.^32^ suggested that subclinical ILD may not significantly affect Grade 2–5 RP or outcomes, though rare cases of extensive RP still occurred.

Patients diagnosed with both IPF and lung cancer exhibit a higher risk of all-cause mortality compared with those with IPF alone (HR: 1.51, 95% CI: 1.22–1.86; *p* < 0.0001).^33^ Similarly, individuals with concurrent IPF and lung cancer demonstrate increased all-cause mortality compared with IPF patients without cancer (HR: 2.39, 95% CI: 1.61–3.54; *p* < 0.0001).^33^ IPF patients are also at greater risk of complications from diagnostic biopsy and cancer therapy. RP and subsequent radiation fibrosis following SABR can be particularly severe in ILD and IPF, as highlighted across multiple retrospective series. A Montreal cohort^12^ reported a 21% incidence of Grade 5 RP after SABR, while Takeda et al.^34^ documented 7 cases of RP among 133 treated patients, including 2 in IPF. Onishi et al.^8^ similarly reported a 6% rate of Grade 5 toxicity at 3 years in IPF patients, compared with a treatment-related mortality of 0.6% among unselected SABR cohorts.

Chen et al.^3^ performed a systematic review of IPF patients treated for NSCLC. SABR-related mortality was 33% in studies including only IPF patients versus 14% in broader cohorts (*p* = 0.092), while treatment-related toxicity differed significantly (71% vs 22%, *p* = 0.01). Among SABR-treated patients, the weighted proportion of ILD-specific mortality was 7.3% in studies with rigorous radiologic and pathologic ILD assessment versus 22.4% in all other studies (*p* = 0.025). For SABR specifically, treatment-related mortality was 15.6% and morbidity 25%. Importantly, studies incorporating both radiologic and pathologic ILD characterisation before treatment reported significantly lower treatment-related mortality (7.3% vs 22%), underscoring the importance of thorough pretreatment evaluation.^35^

Taken together, patients with ILD, and particularly those with IPF, remain at markedly increased risk from SABR. Across published series, RP rates are consistently higher than in non-ILD cohorts, with absolute risks of approximately 20%–50% for Grade ⩾2 RP, 10%–40% for Grade ⩾3 RP and 5%–20% for fatal events (Grade 5) among ILD patients, with the greatest risks in IPF or F-ILD patterns. Multiple large retrospective and prospective studies^12,22,28–30^ demonstrate 3- to 10-fold increases in RP incidence across all severity levels in ILD patients, along with significantly reduced OS. Although mild or subclinical ILD may carry comparatively lower risk, patients with advanced or F-ILD remain at high risk of severe or life-threatening toxicity. IPF represents the highest-risk subgroup, with SABR-related mortality ranging from 15% to 33% and severe toxicity from 25% to 71%, particularly in rigorously phenotyped cohorts.^3,35^ Collectively, these data emphasise that the absolute risk of Grade ⩾2 RP is approximately 20%–50% in ILD and 30%–70% in IPF, far exceeding that of non-ILD populations and highlighting the need for meticulous pretreatment ILD evaluation, careful risk stratification and individualised clinical decision-making.

## Mechanistic plausibility

**Figure 1.**
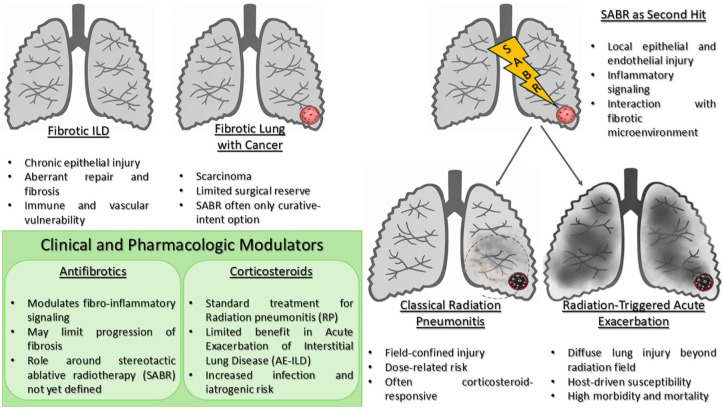
Conceptual model of RT-AE in fibrotic interstitial lung disease. Source: Figure 1 was created by the authors for this article. RT-AE, radiation-triggered acute exacerbation.

The pathobiology of RT-AE in F-ILDs is likely multifactorial, representing a convergence of host-specific vulnerability, radiation-induced immune activation and impaired repair capacity. Clinically, these events often correspond to the so-called ‘sporadic’ form of RP, an unpredictable, occasionally fatal response that may arise even outside the high-dose treatment field and disproportionately affects patients with pre-existing interstitial abnormalities.^12,36^ While causality cannot be established from current evidence, several converging mechanisms may explain why a subset of patients with fibrotic lungs experience catastrophic respiratory decline after SABR. Radiation injury initiates a complex sequence of epithelial and endothelial damage, inflammatory cytokine release and fibroblast activation, culminating in alveolar-capillary barrier disruption.^15,16^ Within hours of exposure, reactive oxygen species and DNA strand breaks activate Nuclear Factor kappa-light-chain-enhancer of activated B cells (NF-κB), Interleukin-1β (IL-1β), Interleukin-6 (IL-6), Tumour Necrosis Factor-α (TNF-α) and Transforming Growth Factor-β (TGF-β) signalling, up-regulating profibrotic mediators such as connective-tissue growth factor and platelet-derived growth factor (PDGF).^25,26^ In most individuals, this inflammatory cascade resolves; however, in fibrotic lungs already characterised by aberrant epithelial-mesenchymal crosstalk and defective regenerative signalling, radiation may amplify an ongoing wound-healing programme, converting a localised insult into a diffuse fibro-inflammatory crisis.^4,13^ The ‘two-hit’ hypothesis provides one conceptual framework: a chronically primed fibrotic lung (first hit) undergoes acute alveolar stress from radiation (second hit), leading to exaggerated cytokine release and diffuse alveolar damage. Pathologic studies reveal that both AE-ILD and high-grade RP share the same histologic substrate: diffuse alveolar damage with hyaline membranes and fibroblast foci.^18,19^ Clinical evidence reinforces this overlap: even patient-adapted robotic SABR with stringent dose constraints and acceptable lung V20 has produced fatal pneumonitis in individuals with IPF, underscoring that intrinsic disease biology rather than dosimetry alone determines catastrophic outcomes.^37^ The distinction may therefore lie not in pathology but in spatial extent and systemic amplification. In RT-AE, local radiation injury could spill over through endothelial activation, circulating cytokines or extracellular vesicles to trigger diffuse alveolar injury beyond the irradiated field. Endothelial dysfunction and microvascular injury appear central to this amplification. Micro-CT and radiobiologic studies demonstrate capillary rarefaction and sustained vascular leakage after thoracic irradiation, which may predispose to hypoxia-driven fibroblast proliferation and release of vascular endothelial growth factor (VEGF).^24,38^ In patients with IPF, telomere shortening and cellular senescence further limit the reparative potential of alveolar epithelial cells,^39^ suggesting that radiation may simply accelerate an already failing repair programme. The immunologic milieu adds another layer of complexity. SABR is known to induce both pro-inflammatory and immune-modulatory effects, sometimes exploited therapeutically in the ‘abscopal effect’ when combined with immune checkpoint inhibitors.^40^ In fibrotic lungs, however, this immune perturbation may become maladaptive. Up-regulation of type-I interferon pathways and macrophage polarisation toward an M2 phenotype could intensify fibroblast recruitment and matrix deposition. Similarly, radiation-induced lymphopenia, a common on-target consequence of high-dose therapy, may suppress protective immune surveillance, predisposing to latent infection or reactivation; events long recognised as AE triggers.^41,42^ Pharmacologic interactions may further modulate this response. Antifibrotic agents such as nintedanib and pirfenidone inhibit key pathways (VEGF, Fibroblast Growth Factor: FGF, PDGF and TGF-β) implicated in RP and fibrosis, suggesting a potential protective effect.^43,44^ Conversely, corticosteroids, while standard for classical RP, may exacerbate vulnerability by suppressing host immunity and enabling infection or delayed epithelial repair.^5,6^ Thus, the steroid-radiation-infection triad may underlie many fatal post-SABR deteriorations previously attributed to idiopathic AE. Emerging radiomic and artificial intelligence analyses suggest that radiographic texture heterogeneity and perivascular density on planning CTs may predict radiation-induced lung injury, hinting at quantifiable endophenotypes of susceptibility.^45,46^ Whether these imaging biomarkers reflect subclinical inflammation, vascular fragility or fibrotic stiffness remains uncertain, but they provide a measurable interface between radiobiology and patient phenotype. Ultimately, the mechanistic evidence supports a plausible biological continuum rather than discrete entities. Radiation can serve as both a local tissue toxin and systemic immune trigger in a lung primed for exaggerated injury. Yet these hypotheses remain inferential, drawn from small, often retrospective datasets and indirect experimental analogies. Clarifying whether RT-AE represents an amplified form of RP or a distinct AE-ILD phenotype will require translational studies incorporating cytokine profiling, endothelial biomarkers and longitudinal imaging in well-phenotyped cohorts.^10,47^ Until then, caution dictates viewing radiation-triggered exacerbations as a biologic possibility rather than a proven entity; one that underscores the fragile balance between curative intent and respiratory catastrophe in fibrotic lungs. These converging pathways, and the divergence between localised RP and host-driven RT-AE, are summarised schematically in Figure 1.

### Risk modifiers

For all patients, a history of smoking, chronic obstructive pulmonary disease (COPD), and ILD is associated with an increased risk of RP. Early SABR experience reported by Yamashita et al.^48^ demonstrated a high incidence of fatal RP among patients with poor respiratory function, interstitial pneumonitis or postoperative recurrence, attributed to both patient-related vulnerability and irradiation of large lung volumes. Dose-volume parameters are also critical: Barriger et al.^17^ reported a 9.4% rate of Grade 2–4 RP, with symptomatic RP correlating strongly with MLD and V20. In a retrospective study by Bahig et al.,^12^ lower forced expiratory volume in one second (FEV_1_) and forced vital capacity, higher V5 and MLD, severe radiological ILD, and coexisting emphysema were significant predictors of Grade ⩾3 RP on univariate analysis, although only FEV_1_ remained significant in the multivariate model. Additional work by Kita et al.^38^ identified interstitial pneumonia as an independent risk factor for symptomatic RP and proposed a lung V10 ⩽ 16.7% threshold for SABR, consistent with findings reported by Baker et al.^23^ Central tumour location has also been associated with greater RP risk compared with peripheral lesions, supported by both contemporary analyses identifying central lesions as an independent predictor of symptomatic RP^38^ and the early phase II experience by Timmerman et al.,^49^ where central tumours showed substantially higher rates of severe treatment-related toxicity.

Patients without radiologic evidence of ILD typically exhibit low rates of severe RP following SABR, with Grade ⩾3 events reported in approximately 0%–10% of cases in general early-stage NSCLC cohorts. In contrast, across ILD-enriched cohorts, severe RP occurs substantially more often, with Grade ⩾3 events reported in approximately 6%–22%, and Grade 4–5 toxicity, although still uncommon, is documented particularly in those with fibrotic changes, like honeycombing.^8,29^ Across studies, dose-volume metrics such as V5–V25 and MLD, as well as biomarkers like Krebs von den Lungen-6 (KL-6), have been associated with RP risk. Additional candidate risk markers include elevated circulating mediators such as TGF-β1, IL-6, KL-6, surfactant proteins and IL-1 receptor antagonist, with KL-6 elevated in more than 70% of affected cases in some cohorts.^50^ However, these biomarkers remain exploratory, with heterogeneous assays and limited prospective validation. Coexisting heart disease has been suggested as a potential contributor to fatal RP, although available evidence remains limited.^8^

Increased pulmonary fludeoxyglucose (FDG) uptake on positron emission tomography (PET) imaging, reflecting heightened inflammatory or metabolic activity, may also predict susceptibility to RP and serve as an early biomarker of subclinical vulnerability prior to SABR. Taken together, these findings highlight that while dose-volume parameters retain prognostic value, the presence and severity of ILD repeatedly emerge as dominant determinants of high-grade RP, underscoring the primacy of host biology over dosimetric thresholds alone.

Smoking history warrants explicit consideration as a background modifier in this context. Cigarette smoking is a shared risk factor for lung cancer and chronic parenchymal lung injury, frequently coexisting with emphysema and F-ILD, and may therefore contribute indirectly to reduced pulmonary reserve and vulnerability to respiratory decompensation. However, available data do not support smoking as a specific or independent trigger of RT-AE following SABR. Rather than acting as a direct causal factor, smoking status likely reflects cumulative lung injury and host susceptibility and should be interpreted as a contextual modifier rather than a mechanistic driver of RT-AE.

### Therapeutic interactions and contested areas

Radiotherapy is a fundamental component of lung cancer treatment and is generally well tolerated, though toxicity risk varies with dose, irradiated lung volume and anatomical location.^15,34^ Management of symptomatic RP typically involves corticosteroids, but they do not reliably prevent progression to fibrosis and their benefit remains uncertain.^6^ Severe RP is usually treated with systemic corticosteroids followed by a gradual taper,^15^ and when treatment is expected to exceed 1 month, Pneumocystis jirovecii prophylaxis is advised^42,51^; depending on the degree and duration of immunosuppression, broader infection prophylaxis may also be necessary.^52^ Steroid-refractory RP, often accompanied by elevated KL-6 levels, may respond to immunosuppressive agents such as azathioprine or cyclosporine.^15^

Despite corticosteroids being considered the standard of care for RP, their application in patients with ILD, particularly IPF, requires caution.^53–55^ Evidence supporting corticosteroid efficacy in AE-IPF remains limited and inconsistent. A retrospective cohort study by Farrand et al.^56^ demonstrated no benefit of corticosteroids in AE-IPF, although two registered prospective trials (NCT05674994 and NCT04996303) are currently underway, with no published results to date. Current IPF guidelines offer only a weak recommendation for corticosteroids in AE-IPF and provide no guidance on optimal dosing or duration, and importantly, they do not address RT-AEs at all.^4^

Accurately distinguishing RP from alternative causes of acute respiratory deterioration, including infection, heart failure, drug-induced lung injury, lymphangitic carcinomatosis and tumour progression, remains challenging in lung cancer populations.^57^ Because of this diagnostic uncertainty, patients presenting with abrupt worsening dyspnoea are frequently treated empirically with broad-spectrum antibiotics and corticosteroids. Multidisciplinary input from pulmonology, radiation oncology and thoracic radiology may improve diagnostic precision before initiating immunosuppressive therapy, particularly relevant when evaluating possible RT-AE in patients with underlying F-ILD.^58^

Importantly, no robust randomised evidence confirms long-term clinical benefit from corticosteroids in RP, nor is there validation of steroid efficacy once fibrosis has evolved. While corticosteroids remain the mainstay of treatment for symptomatic or progressive RP, high-quality clinical trials are urgently needed, especially given their uncertain benefit and potential harm in F-ILD.^55^ Emerging therapeutic approaches include the multi-kinase inhibitor nintedanib, which targets VEGF and related profibrotic pathways. Phase II data^44^ suggest that nintedanib combined with a prednisone taper may reduce pulmonary exacerbations over 1 year, although further investigation is required. Pirfenidone has also shown promise in radiation-induced lung injury, with retrospective data^43^ demonstrating improved outcomes in NSCLC patients with grade ⩾2 toxicity treated with pirfenidone plus a short corticosteroid taper. Most prophylaxis strategies studied to date originate from surgical IPF literature rather than oncologic settings. In perioperative cohorts, trials evaluating methylprednisolone with sivelestat or ulinastatin showed acceptable safety profiles, and pirfenidone was associated with a reduced incidence of postoperative AE-IPF (3.2% vs 21.1%).^59^ Nintedanib likewise reduced AE risk in the broader IPF population.^60,61^ However, no prophylactic strategies have been comparably evaluated in SABR-treated patients, and extrapolation to the radiotherapy context remains speculative. Although corticosteroids are widely used for RP, controlled studies do not support their effectiveness in preventing or treating AE-IPF,^3,5,6,57^ underscoring the need for caution when applying RP paradigms to RT-AE.

Interactions between radiotherapy and immune checkpoint inhibitors (ICIs) add further complexity. Li et al.,^62^ in a systematic review and meta-analysis of pneumonitis among patients receiving ICIs in combination with radiotherapy or chemoradiotherapy, found that conventional radiotherapy was associated with higher pneumonitis rates than stereotactic approaches, with all-grade pneumonitis occurring in 37% versus 26% and severe (grade ⩾3) pneumonitis in 3% versus 1%, respectively. Sequential immunotherapy was linked to higher rates of all-grade pneumonitis compared with concurrent administration, and anti-programmed cell death protein 1 (PD-1) agents carried a greater pneumonitis risk than programmed death ligand 1 (PD-L1) inhibitors. These findings highlight the potential for immune-amplified lung injury when ICIs are combined with thoracic irradiation, a particular concern in patients with F-ILD undergoing SABR and subsequent systemic therapy.

No formal guidelines exist for corticosteroid therapy in RP occurring in patients with preexisting ILD. Management, therefore, relies largely on expert opinion. Evidence supporting steroid efficacy is limited to retrospective studies, and no society-level recommendations specifically address RP in patients with underlying ILD. Given that patients with F-ILD can be particularly prone to severe inflammatory flares and often show incomplete responses to corticosteroids, dedicated research and population-specific management frameworks are urgently needed.

### Pragmatic considerations and shared decision-making

Multidisciplinary care with coordinated multidisciplinary team meetings and clinics enhances collaboration, clarifies team roles and supports high-quality, guideline-based lung cancer management, potentially improving survival and patient quality of life.^63^ Radiation-induced lung injury after SABR ranges from early RP, within 6 months, to late radiation fibrosis, with radiological signs typically appearing around 17 weeks but sometimes delayed up to a year; diagnosis can be challenging in patients with pre-existing lung conditions such as COPD or ILD.^64^ Serial CT scans, complemented by FDG-PET, preferably 6 months after SABR,^65^ and pathological confirmation when available, remain the standard for detecting local recurrence, with surveillance schedules guided by major guidelines and expert consensus, generally every 3–6 months in the first 2 years, then annually through year 5, with closer monitoring for high-risk patients, including those with ILD.^10^ In F-ILD, post-SABR imaging interpretation is particularly challenging because RP, RT-AE, infection and progression of underlying fibrosis may exhibit overlapping CT patterns.^26^

SABR is the preferred treatment for medically inoperable or surgery-declining early-stage NSCLC, achieving over 90% local control with low severe toxicity and minimal mortality, making it suitable even for frail patients. Patients with ILD are at higher risk of SABR-related toxicity, yet options remain limited. The ASPIRE-ILD trial demonstrated that SABR more than doubled median survival in this population and showed lower severe toxicity than previously reported, likely due to careful planning, dosing and follow-up.^10^ Shared decision-making should explicitly address the possibility of RT-AE, which may occur unpredictably and can be catastrophic even when dosimetric constraints are respected.^66^

Alternative curative approaches, such as surgery, thermal ablation or observation, may be considered, though each carries a higher risk or uncertain benefit. Assessing ILD severity using the ILD-GAP Index, which accounts for ILD type, sex, age and lung function, helps guide suitability and dosing for SABR.^10^ Finally, a baseline high-resolution chest CT is essential before SABR, as it helps assess a patient’s risk of developing RP and may distinguish RP from an exacerbation of an unknown pre-existing ILD.

### Limitations

Most of the studies included in this review were retrospective, introducing inherent selection and publication bias, as cohorts demonstrating more striking outcomes were more likely to be reported. Substantial heterogeneity existed in study design, objectives and endpoint definitions. Some investigations explicitly evaluated the impact of ILD on radiotherapy-related toxicity, whereas others described ILD only incidentally within larger oncologic cohorts. This variability complicates cross-study comparison and may contribute to overestimation of toxicity risk among individuals with F-ILD. Terminology inconsistencies further limit interpretability. Many reports used nonspecific descriptors such as interstitial pneumonitis or idiopathic interstitial pneumonia interchangeably with radiopathologic terms like UIP or nonspecific interstitial pneumonia, often without multidisciplinary confirmation or standardised diagnostic criteria.^4^ Dosimetric reporting was also heterogeneous, with differences in lung contouring, selected Vx cut-offs and MLD thresholds across series, limiting formal dose-response modelling and cross-study comparison.^17,23,24^ The evidence base is geographically skewed, dominated by East Asian cohorts where ILD prevalence, imaging thresholds and management strategies may differ from Western practice.^7,8,22,31^ In addition, many cohorts span nearly two decades, during which SABR techniques evolved from fixed-frame to image-guided and robotic platforms, introducing temporal bias and further reducing comparability of toxicity estimates.^2,25,33^ Few studies included histopathologic or biomarker correlates, and almost none incorporated antifibrotic exposure, telomere length or cytokine profiling. Baseline and longitudinal pulmonary function trajectories were rarely reported, leaving uncertainty about pre-existing impairment and post-SABR pneumonitis recovery patterns. Furthermore, most cohorts aggregated heterogeneous ILD phenotypes without subgroup analysis, thereby obscuring phenotype-specific risk signals and limiting clinical translation. Mechanistic interpretations presented in this review remain hypothesis-generating. While radiation may plausibly act as a trigger of AE through epithelial and endothelial injury, immune activation and microvascular dysfunction, these mechanisms have not been prospectively validated. Consequently, causality cannot be inferred, and the biological continuum proposed between RP and AE-ILD should be regarded as conceptual rather than definitive. Together, these limitations underscore the need for prospective, multicenter studies employing standardised ILD classification, uniform SABR dosimetry reporting and integrated translational endpoints to define risk boundaries more accurately and develop targeted mitigation strategies.

### Future directions

Progress in managing lung cancer within F-ILD will depend on moving from retrospective description toward prospective, mechanistic investigation. Existing studies are largely confined to East Asian series with heterogeneous case definitions and limited translational endpoints. Multinational collaborations, ideally embedded within ILD and oncology networks in Europe and North America, are needed to characterise RT-AEs as a discrete, post-therapeutic phenotype rather than an anecdotal complication.^66,67^ Clinically, the next step is a prospective registry capturing detailed radiotherapy parameters, ILD phenotype and short-term outcomes. Such a dataset would enable risk-stratified modelling beyond the current empirical thresholds and clarify whether the apparent loss of dose-response for grade ⩾3 events truly reflects stochastic host susceptibility.^17,23,38^ Integration of pulmonary function trends, antifibrotic exposure and bronchoalveolar or serum biomarkers could enable the development of multidimensional pre-SABR risk signatures that move beyond morphologic UIP patterning alone. To this end, forthcoming studies should embed systematic pulmonary function testing to capture the physiologic trajectory of the irradiated fibrotic lung and to link functional decline with radiographic and clinical outcomes. Parallel efforts must focus on refined phenotyping of ILD subgroups, thereby permitting phenotype-specific modelling of radiation vulnerability. Such integration of functional, morphologic and biologic dimensions will provide the foundation for truly personalised risk assessment and targeted mitigation strategies and will also create a translational framework to interrogate the immuno-fibrotic axis linking RP and AE-ILD. Profiling cytokine kinetics and fibroblast activation pathways pre- and post-SABR could test whether these entities share common effector cascades and identify pharmacologic points of convergence. At present, there is also insufficient evidence to support an association between tumour molecular characteristics and the risk of RT-AE in F-ILD, highlighting an area for future research. Likewise, telomere biology and markers of epithelial senescence may stratify vulnerability to radiation injury.^39^ From a therapeutic perspective, antifibrotic-radiation interaction studies are urgently required. Preliminary observations suggest that continued nintedanib or pirfenidone is feasible and may mitigate downstream fibrosis, but confirmatory data are lacking.^43,44^ Trials testing antifibrotic co-therapy or peri-radiation intensification should incorporate infection surveillance and avoid uncontrolled steroid exposure. Parallel evaluation of particle therapies (proton or carbon-ion) in UIP-pattern disease could determine whether their favourable physical dose distributions translate into clinically meaningful toxicity reduction.^68,69^ Finally, multidisciplinary research platforms linking radiation oncologists, pulmonologists, radiologists and immunologists should standardise terminology, imaging criteria and biospecimen protocols. Only through such coordinated efforts can the field progress from cautionary anecdote to evidence-based precision care, transforming RT-AE-ILD from an unpredictable hazard into a potentially preventable or modifiable event.

## Conclusion

Radiation therapy remains central to the curative management of early-stage lung cancer, yet patients with F-ILD face a disproportionately high risk of severe respiratory decline after SABR. Accumulating evidence suggests that a meaningful subset of post-SABR ‘pneumonitis’ episodes does not conform to classical, dose-confined, dose-dependent RP, but instead resembles RT-AE of the underlying fibrotic disease, reflecting host vulnerability in a biologically primed lung. This framing matters clinically: current practice often defaults to corticosteroids, despite limited evidence of benefit in AE-ILD and a plausible potential for harm in progressive fibrotic disease, particularly through infectious and iatrogenic complications. Antifibrotic agents hold mechanistic promise, but their role around radiotherapy remains insufficiently defined. Until higher-quality evidence is available, SABR should be delivered within a multidisciplinary framework that explicitly addresses the possibility of RT-AE, balances curative intent against respiratory risk, and anchors decisions in individualised, transparent shared decision-making. Future progress will require prospective registries with standardised ILD phenotyping and dosimetric reporting, integrated functional and biomarker endpoints, and pragmatic trials testing antifibrotic-radiation strategies and particle therapies in carefully selected high-risk populations.

## Supplemental Material

sj-docx-1-tar-10.1177_17534666261446877 – Supplemental material for Radiation-triggered acute exacerbation of progressive fibrotic interstitial lung diseases: ‘Are we advancing the frontier or crossing a dangerous line?’ Supplemental material, sj-docx-1-tar-10.1177_17534666261446877 for Radiation-triggered acute exacerbation of progressive fibrotic interstitial lung diseases: ‘Are we advancing the frontier or crossing a dangerous line?’ by Ilias E. Dimeas, Angeliki Miziou, Patrick D. Mitchell, Zoe Daniil and Cormac McCarthy in Therapeutic Advances in Respiratory Disease

## Declarations

**Ethics approval and consent to participate:** Not applicable. This study is a narrative review based exclusively on previously published literature and did not involve human participants, patient data or animal experiments.

**Consent for publication:** Not applicable. This article does not contain any individual patient data or identifiable information.

**Availability of data and materials:** No new data were generated or analysed in this study. All information discussed in this article is derived from previously published literature cited in the reference list.
