# Supplementary material for: Radiation-triggered acute exacerbation of progressive fibrotic interstitial lung diseases: ‘Are we advancing the frontier or crossing a dangerous line?’
Source: Ther Adv Respir Dis. 2026 May 5;20:17534666261446877. doi: 10.1177/17534666261446877 (PMC13161628; doi:10.1177/17534666261446877)
Supplement: sj-docx-1-tar-10.1177_17534666261446877 – Supplemental material for Radiation-triggered acute exacerbation of progressive fibrotic interstitial lung diseases: ‘Are we advancing the frontier or crossing a dangerous line?’ [file sj-docx-1-tar-10.1177_17534666261446877.docx]

# SANRA Checklist: Narrative Review Submission

Manuscript: Radiation-triggered acute exacerbation of progressive fibrotic interstitial lung diseases: “Are We Advancing the Frontier or Crossing a Dangerous Line?

Each item is scored from 0 (not at all) to 2 (fully addressed).

| Item | Assessment | Score (0–2) |
| --- | --- | --- |
| Justification of the article’s importance for the readership | The manuscript explores radiation-triggered acute exacerbations in fibrotic ILDs; a lethal but under-recognised complication at the interface of interstitial lung disease and radiation oncology. This topic has direct implications for the management of patients with inoperable lung cancer and fits the Special Issue “When the Air Gets Rare: Advances in Rare Lung Disease Management.” | 2 |
| Statement of concrete aims or formulation of questions | The stated aim is to synthesize available evidence on the risk, pathophysiology, and management controversies surrounding acute respiratory events following stereotactic ablative radiotherapy (SABR/SBRT) in progressive fibrotic ILDs, and to generate hypotheses for future trials. | 2 |
| Description of the literature search | A scoping narrative search of PubMed and Embase (2000–2025) was conducted, including 65 PubMed-indexed English-language studies covering AE-ILD definitions, radiation pneumonitis, dosimetric predictors, antifibrotic therapy, and particle modalities. Both clinical and mechanistic papers were incorporated; no PRISMA approach was applied given the hypothesis-generating intent. | 2 |
| Referencing | PubMed-indexed references were used, spanning 2000-2025, incorporating recent high-impact studies. All statements are evidence-anchored and referenced in Vancouver style. | 2 |
| Scientific reasoning | The manuscript critically analyses existing data, identifies methodological gaps (retrospective bias, geographic skew), and challenges reflexive high-dose steroid use. It develops the hypothesis of a radiation-triggered AE-ILD phenotype driven by immune dysregulation and infection risk, balancing clinical caution with oncologic necessity. | 2 |
| Appropriate presentation of data | The article follows a structured flow (Introduction → Methods → Diagnostic Ambiguity → Risk → Mechanisms → Modifiers → Therapeutic Interactions → Pragmatic Considerations → Future Directions → Conclusion). | 2 |

Total Score: 12 / 12
